# Point-of-Care Ultrasound to Locate Retained Intravenous Drug Needle in the Femoral Artery

**DOI:** 10.5811/westjem.2016.8.31074

**Published:** 2016-09-12

**Authors:** Blake Primi, Molly E.W. Thiessen

**Affiliations:** * University of Colorado School of Medicine, Department of Emergency Medicine, Aurora, Colorado; † Denver Health Medical Center, Department of Emergency Medicine, Denver, Colorado

## Abstract

We describe the use of point-of-care ultrasound to localize a retained intravenous drug needle, and subsequent surgical removal without computed tomography.

## CASE

A 33-year-old male presented to the emergency department (ED) with left groin pain. Six days prior, a needle had broken off in his groin while injecting intravenous (IV) drugs. On exam, he had track marks in his left groin, but no evidence of infection. The neurovascular exam of his left lower extremity was normal.

The patient had a point-of-care ultrasound (POCUS) initially, and subsequently a plain film of his left groin.

**Figure 1:**
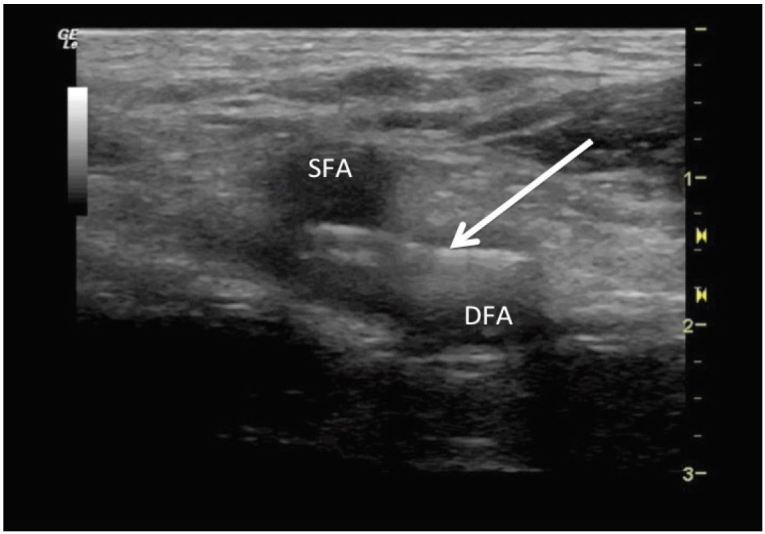
Linear foreign body (arrow) within the femoral artery just distal to the bifurcation of the superficial femoral artery (SFA) and the deep femoral artery (DFA), consistent with retained needle, as seen on point-of-care ultrasound.

The POCUS of his left groin demonstrated a linear foreign body oriented horizontally through his superficial femoral artery and deep femoral artery, just distal to the bifurcation. (Video 1, Figure 1)

**Figure 2:**
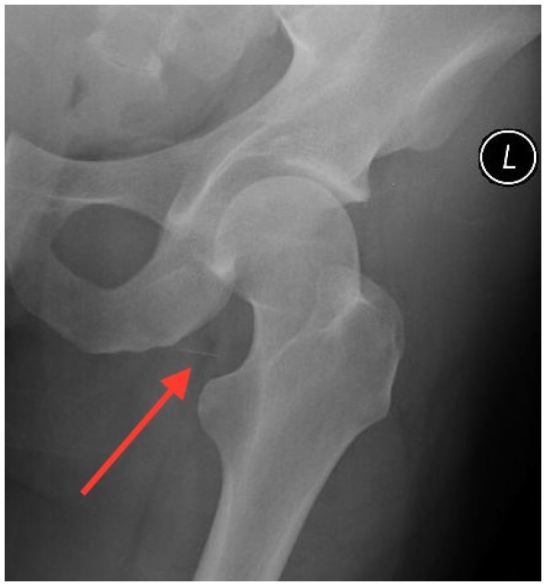
Linear foreign body (arrow) in the groin as seen on plain radiograph.

A plain radiograph confirmed these findings (Figure 2).

The patient was taken from the ED to the operating room (OR) with no additional imaging. In the OR, the surgical team confirmed the presence of the foreign body with fluoroscopy, then dissected down to the femoral artery. Using the anatomic landmarks described in the POCUS, the surgery team localized and removed the needle. The patient was discharged later that morning.

## DISCUSSION

Needle loss is not a rare occurrence for IV drug abusers.^1^,^2^ When dislodgement occurs in the vasculature, grave complications can ensue, as the needle has the potential to embolize to the right heart or lungs. Prompt extraction is therefore necessary.^3^,^4^ Surgical extraction typically requires a pre-procedural computed tomography (CT) to localize the object.^5^,^6^ While effective, CTs are costly, expose the patient to considerably high doses of radiation, and lengthen the time to definitive treatment. Ultrasound is a well established method of locating radiolucent foreign bodies,^7^,^8^ with comparable efficacy in the detection of radiopaque foreign bodies in soft tissue when compared to CT.^9^,^10^ In cases of smaller wooden splinters, it has been found to be superior to CT.^11^ In this case, we described the use of POCUS to localize a retained IV drug needle that was then surgically removed without complication, emphasizing the value of POCUS as a timely, cost-saving, radiation-sparing technology.

## Figures and Tables

**Video 1:** Video of point-of-care ultrasound demonstrating the linear foreign body, consistent with retained needle, oriented horizontally just distal to the bifurcation of the superficial femoral artery (SFA) and the deep femoral artery (DFA).
